# Cytokine Changes in Gingival Crevicular Fluid and Serum After Non-Surgical Periodontal Therapy in Patients with Periodontitis: A Systematic Review and Narrative Synthesis

**DOI:** 10.3390/jcm14238424

**Published:** 2025-11-27

**Authors:** Christine J. H. Kim, Matthew Baker, Carlos Marcelo S. Figueredo

**Affiliations:** 1 School of Medicine and Dentistry, Griffith University, Gold Coast, QLD 4215, Australia; christinejh.kim@griffithuni.edu.au (C.J.H.K.); m.baker1@griffith.edu.au (M.B.); 2 Division of Oral Diseases, Department of Dental Medicine, Karolinska Institute, 171 77 Stockholm, Sweden

**Keywords:** periodontitis, NSPT, cytokines, Th-17

## Abstract

**Objective:** We aimed to synthesize paired pre/post human evidence on how Th17-axis cytokines (IL-17A, IL-21, IL-22, IL-23 and related markers) change after non-surgical periodontal therapy (NSPT) by biospecimen (gingival crevicular fluid [GCF], saliva, serum) and time window. **Material and methods:** We performed a PRISMA-guided systematic review of non-randomized pre/post cohorts and clinical trials. Databases (PubMed, Embase, Scopus, Cochrane) were searched; two reviewers performed selection, data extraction, ROBINS-I risk-of-bias appraisal, and GRADE certainty assessment. Due to heterogeneity in sampling/assays and incomplete variance reporting, a qualitative direction-of-effect synthesis was prespecified for ≤4 weeks, ~6–8 weeks, and ~3–6 months. **Results:** Twelve studies (total n = 897) met inclusion (8 GCF; 5 blood-derived (serum/plasma) cohorts; one saliva). Most GCF cohorts reported decreases in IL-17A within ~6–8 weeks post-NSPT (≥4 cohorts), with one early 4-week cohort showing a concentration increase but an unchanged total amount due to reduced GCF volume. IL-23 generally declined locally and declined by ~3 months systemically in aggressive periodontitis. Serum IL-17A changes were smaller/variable (two cohorts reported decreases within 1–6 months), and one cohort showed a reduced IL-17A:IL-17E ratio at ~25 weeks. Heterogeneity precluded meta-analysis; we undertook a direction-of-effect synthesis. **Conclusions:** NSPT is likely associated with the early local down-regulation of Th17-axis activity (notably GCF IL-17A), when systemic signals are smaller and delayed. Given moderate–serious risk of bias and pre-analytical heterogeneity, the certainty of evidence is low to very low; Th17-axis biomarkers are not yet suitable for clinical decision-making.

## 1. Introduction

Periodontitis is a host-mediated inflammatory disease initiated by a dysbiotic subgingival biofilm and characterized by the progressive loss of periodontal support [1,2,3,4,5]. Beyond single-pathogen models, dysregulated host–microbe interactions drive tissue destruction. The Th17 axis—particularly IL-17A/F, IL-21, IL-22, and IL-23—has been implicated in disease activity and osteoimmunological pathways in human gingiva and oral fluids [6,7,8,9,10,11,12,13,14]. Importantly, results that differ by population and assay illustrate considerable variability; for instance, GCF IL-17A may not be detectable in certain groups, highlighting detection limits and issues with pre-analytical variability [15,16]. In this review, we treat IL-17A, IL-17F, IL-21, IL-22 and IL-23 as a single Th17-associated functional axis because they are co-regulated by Th17 cells and act together to drive neutrophil recruitment, mucosal barrier responses, and RANKL-mediated osteoclastogenesis.

The 2018 framework classifies periodontitis into stages (I-IV) and grades (A-C) based on clinical attachment loss, periodontal pockets, and radiographic bone loss [17,18]. Management begins with non-surgical periodontal therapy and subsequent re-evaluation, progressing to adjunctive or surgical interventions and continued supportive maintenance as necessary [18,19].

Biologically, Th17/IL-17 signaling drives RANKL-mediated osteoclastogenesis, linking inflammation to alveolar bone loss [20,21,22]. Consistent with this, Th17-axis cytokines often decline after NSPT, though magnitudes vary by biospecimen, assay, and follow-up window [23,24,25,26]. The interpretation of GCF cytokines must consider sampling and volume normalization because concentration and total amount can diverge when crevicular fluid flow changes [27,28,29].

Non-surgical periodontal therapy (NSPT) aims to reduce biofilm coverage and may affect Th17-axis signaling; however, studies report inconsistent cytokine outcomes after NSPT [15,17,18]. Variability in patient types, biospecimens, target analytes, assay methods, and follow-up times leads to heterogeneity [15,18,19,20].

In addition, pre-analytical factors—especially whether GCF *volume* is captured—can invert concentration-based interpretations and obscure biologically meaningful changes in total amount [30,31]. Systemically, some cohorts report a reduction in the serum IL-17A:IL-17E (IL-25) ratio by ~25 weeks after NSPT, suggesting a counter-regulatory shift that may accompany clinical improvement [8,9,10,11,12]. In general, earlier syntheses have rarely limited their inclusion to paired pre/post human cohorts or divided results according to compartment and time window, reducing how well the findings can be interpreted [19,26].

We conducted a PRISMA-guided qualitative, direction-of-effect synthesis of Th17-axis cytokines after NSPT in adults. The primary endpoint was the direction of change in GCF IL-17A at ~6–8 weeks. Secondary endpoints included IL-23, IL-21, IL-22, and serum/saliva measures across prespecified windows (≤4 weeks; ~6–8 weeks; ~3–6 months). Where studies reported both concentration and total amount with explicit GCF volume capture, total amount was prioritised. Study quality and certainty were summarised using ROBINS-I and GRADE, respectively; no meta-analysis was planned due to expected heterogeneity.

## 2. Materials and Methods

### 2.1. Study Design

Prospective pre–post human cohorts or clinical trials in English were included. Exclusions included animal/in vitro studies, surgical-only therapy, case series, non-peer-reviewed papers, and peri-implant studies without separate periodontal data. SRP ± adjunct trials were only included when SRP-alone results were available or when adjuncts did not alter the direction of SRP effects; such data were stratified accordingly. We performed the PRISMA-2020-guided systematic review of non-randomized pre/post studies with qualitative direction-of-effect synthesis (no meta-analysis); review-level randomization/allocation and power calculations were not applicable.

### 2.2. Focus Question

How does non-surgical periodontal therapy (NSPT) modulate Th17-axis cytokine profiles in humans in terms of different biological compartments, and what is the trajectory of these changes over time?

### 2.3. Population

We included adults (≥18 years) that were clinically diagnosed with periodontitis based on established parameters, including probing depth and clinical attachment loss; these were categorized according to either the historical chronic/aggressive classification or the 2018 periodontitis staging and grading framework.

### 2.4. Comparator

We used baseline (pre-NSPT) measurements, obtained from the same participants or periodontal sites prior to therapeutic intervention.

### 2.5. Outcome

We assessed post-NSPT alterations in Th17-axis cytokines—specifically IL-17A, IL-21, IL-22, and IL-23—within gingival crevicular fluid, saliva, or serum/plasma, along with related molecular markers such as RORC and the sRANKL/OPG ratio. When both cytokine concentrations and total amounts were reported, priority was given to total quantities that accounted for sample volume. Outcomes up to 6 months were considered.

### 2.6. Search Strategy

The current systematic review was performed in accordance with PRISMA 2020 guidance [32], and the completed PRISMA 2020 checklist is provided in Supplementary Table S6. A protocol was developed a priori (not registered). We did not register the protocol in PROSPERO because, at the time of planning, institutional procedures focused on an internally agreed written protocol and the review progressed rapidly to full-text screening; retrospective registration was considered once data extraction had begun, but we instead prioritised making the protocol and full search strategy publicly available in the Supplement. To enhance transparency and reproducibility, we also documented the complete database search strings and deduplication logic (Supplementary Material S1). Electronic searches and data extraction were performed independently by two reviewers using a standardized template, and all discrepancies were resolved by consensus.

PubMed, Embase, Scopus, and Cochrane Library databases were searched for articles published until 27 August 2025. The search used the following combination of terms: (periodontitis OR periodontal disease) AND (scaling OR root planing OR subgingival debridement OR non-surgical periodontal therapy) AND (Th17 OR IL-17 OR IL-21 OR IL-22 OR IL-23 OR cytokine OR biomarker). No date limits were applied. The reference lists of retrieved papers were hand-searched to identify additional eligible studies. Any inter-examiner disagreements regarding article inclusion were resolved through discussion until consensus was reached. Grey literature was not searched; the review was limited to peer-reviewed articles identified in the listed databases and by hand-searching reference lists.

### 2.7. Selection Criteria

Inclusion criteria: Prospective pre–post cohorts or trials published in English were selected. Exclusion criteria: Animal/in vitro, surgical-only interventions, case reports/series, and non-peer-reviewed publications were selected. Peri-implant disease studies were excluded from this research unless periodontal outcomes were separately reported. For SRP ± adjunct trials, cohorts were only retained if SRP-alone data were available or the adjunct did not change SRP directionality; counts were stratified accordingly. These criteria were chosen to isolate the NSPT-specific immunologic response and avoid heterogeneity from distinct pathologies or interventions.

### 2.8. Intervention

The intervention chosen was non-surgical periodontal therapy (NSPT), involving scaling and root planing with or without standard oral-hygiene instruction and without surgical adjuncts. Adjunctive antimicrobials and laser protocols, when present, were recorded but not analysed as separate interventions unless explicitly reported with stratified cytokine data.

Studies reported at least one Th17-axis mediator (IL-17A, IL-17F, IL-21, IL-22, IL-23, IL-17E) in GCF, saliva, or serum/plasma and/or related readouts (e.g., RORC, sRANKL/OPG). Given volume effects in GCF, we recorded whether studies reported concentration (e.g., pg/mL) or total amount per sampling time/site; when both were available with explicit volume capture, the total amount was prioritized for interpretation; details of GCF volume capture and methods for each study are summarized in Supplementary Table S5.

### 2.9. Methodological Appraisal (Risk of Bias) and Certainty of Evidence

Risk of bias for non-randomized pre/post cohorts was evaluated using ROBINS-I across multiple domains: confounding, selection, classification of interventions, deviations, missing data, outcome measurement, and selective reporting [33]. ROBINS-I criteria were used for judgment, where the highest-risk domain sets the overall risk level; domain-specific reasons are detailed in Supplementary Table S4; GRADE evidence profiles are given in Supplementary Table S3. Since this review focuses on non-randomized pre/post studies, randomization and allocation concealment do not apply at the review level. Any details from randomized study arms were noted and included in the relevant ROBINS-I domains. Certainty for each outcome and area was assessed with GRADE, considering factors such as risk of bias, inconsistency, indirectness, imprecision, and publication bias. Two reviewers independently applied ROBINS-I; any discrepancies were resolved by discussion and consensus between them, and no third reviewer was required.

### 2.10. Data Synthesis (Non-Meta)

Details of sample type (eg GCF, serum), biomarkers analysed, units of measurement, follow-up period, and clinical outcomes (PD, CAL, BOP) were recorded. The primary outcome for biomarkers and clinical outcomes was mean difference following NSPT. Follow-up windows were clustered (≤30 days; ~6–8 weeks; ~3–6 months) to reflect established molecular- and tissue-level healing trajectories after debridement, where early cytokine shifts preceded clinical remodeling.

Primary windows were defined as approximately 6–8 weeks for GCF and 3–6 months for serum. When studies included multiple post-NSPT timepoints, the following hierarchy was applied: (i) the prioritization of data within the primary window; (ii) if data from the primary window were unavailable, the nearest subsequent window was selected; (iii) early data (≤4 weeks) were recorded qualitatively but did not supersede the primary window. For GCF, if there were two outputs and concentrations, and if the total amount was reported with explicit capture volume, the total amount was used for interpretation. Additionally, for all studies, we recorded the preanalytical aspects (e.g., collection device, dwell time, elution, storage, freeze–thaw, and volume capture), as well as how to treat LODs to inform risk of bias and provide a narrative summary for sensitivity.

We did not fit any review-level statistical models or test distributional assumptions (e.g., normality and homoscedasticity) because we did not undertake a meta-analysis. Assays ranged from single-plex ELISAs to multiplex bead panels with different lower limits of detection; primary follow-up windows varied from very early sampling (≤4 weeks) to approximately 6–8 weeks and 3–6 months; and GCF outcomes mixed concentration-only reporting with and without explicit crevicular volume capture. Under these conditions, effect sizes were not comparable on a common scale and key variance estimates were often missing, which precluded valid pooling. For each study, we therefore noted the reported within-study analyses (e.g., paired t tests, Wilcoxon tests, repeated-measures models) and used them solely to confirm the direction of effect, without review-level power calculations or meta-analytic estimation; results are presented descriptively in tables and direction-of-effect summaries.

## 3. Results

### 3.1. Study Selection:

**Figure 1:**
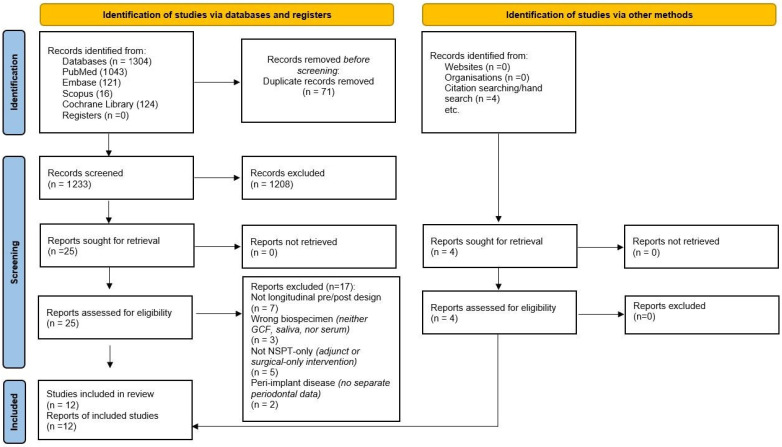
PRISMA 2020 flow diagram of study selection. Note: One included trial (Gür et al., 2022) [39] was an SRP ± adjunct (diode laser) RCT; it is counted once among the 12 included studies, and only the SRP-alone arm contributed cytokine data (adjunct arms excluded).

Study selection is shown below in Figure 1. A total of 1304 total records (PubMed 1043; EMBASE 121; Scopus 16; Cochrane Library 124, as well as 4 articles found through citation screening) were retrieved up to 27 August 2025. Overall, 71 were duplicates and they were removed. Additionally, 29 full texts were obtained from the 1233 titles/abstracts screened, and 17 studies were excluded following full-text reading, primarily for the absence of paired pre- and post-cytokine data or adjunctive treatement elements (Figure 1 and Supplementary Table S2). In total, 12 studies were included in the final review (Table 1) [15,16,23,24,34,35,36,37,38,39,40,41].

### 3.2. Study Characteristics

Key features of the studies included are summarized in Table 1 and Table 2. Most were prospective pre/post cohorts; a minority were clinical trials with SRP ± adjunct arms, from which the SRP-alone arm (or any arm that did not change directionality relative to SRP) was extracted for synthesis. Biospecimens comprised gingival crevicular fluid (GCF), serum, and (in one study) saliva. Follow-up assessments were clustered at ≤30 days, ~6–8 weeks, and ~3–6 months. Assays were predominantly ELISA or multiplex immunoassays. When both concentration and total amount were reported with explicit GCF volume capture, total amount was prioritized.

Most cytokine markers showed significant pre/post decreases (*p* < 0.05 in most cohorts), although a few did not reach the level of statistical significance. (Table 2). The observed declines in important mediators, such as IL-17A and IL-23, were not only consistent but also unlikely to be attributable to chance, as evidenced by *p*-values < 0.05 in most cohorts. This cross-study pattern of considerable cytokine reduction quantitatively validates the overall downward trend identified in our synthesis conclusions.

### 3.3. Results Structure

Local (GCF) outcomes were assessed at ~6–8 weeks and ~3 months, while systemic (se-rum) effects were evaluated at ~1–6 months. For each compartment, we report IL-17A, IL-23, IL-21, and IL-22 levels, and also produce a brief note on counter-regulatory markers and bone coupling readouts. Table 3 highlights the studies’ pre-analytical and analytical details, and Supplementary Table S5 indicates, for each GCF cohort, whether and how crevicular volume was measured. Table 4 shows the Summary of Findings (GRADE) for each outcome, including the compartment, timeframe, direction of effect, number of cohorts, and GRADE certainty.

To quantify within-study changes, Table 2 compiles pre- and post-NSPT values with absolute and percentage differences, author-reported *p*-values (and confidence intervals where available), and a qualitative direction-of-effect label (increase, decrease, no significant change, or not detectable) for each cytokine.

### 3.4. Risk of Bias

**Figure 2:**
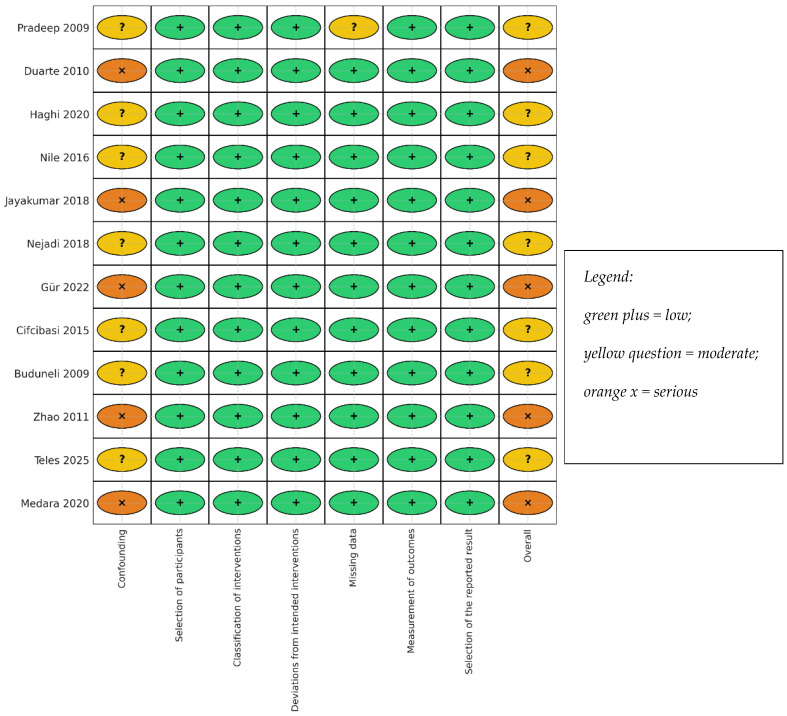
Risk of bias ROBINS-I of the included studies (traffic-light) [15,16,23,24,34,35,36,37,38,39,40,41].

Overall, ROB was moderate or serious, driven by site-within-person clustering, incomplete GCF volume capture, and small samples; no cohort was reported to be at low overall risk. ROBINS-I domain concerns clustered in confounding and outcome measurement (GCF concentration-only reporting without volume capture). Across the twelve cohorts, six were rated as displaying overall “moderate” risk and six as displaying “serious” risk, with none showing “low” risk, consistent with Figure 2. These judgements informed the GRADE ratings: low for GCF IL-17A; low for serum IL-17A/IL-23 and GCF IL-21; and very low for IL-22 and sRANKL/OPG.

### 3.5. Local (GCF) Cytokine Kinetics After NSPT

#### 3.5.1. IL-17A

Most cohorts reported a decline in GCF IL-17A levels approximately 6–8 weeks after NSPT, with suppression consistently observed and continuing for approximately 3 months [23,24,39,40]. In generalised aggressive periodontitis, both local and systemic reductions were consistently observed around three months [34]. Two preliminary caveats should be noted during data interpretation. First, at approximately 4 weeks, the IL-17A levels increased, while their total quantity remained unaltered. These findings are explained by the shrinkage of GCF volume post-therapy. Concentration measurements taken without accounting for volume inflate the values of concentration; therefore, the total quantity is necessary to interpret IL-17A [16]. Second, the detected levels of IL-17A were low in one previous cohort, both before and after therapy. This is mainly attributed to the sensitive limits of the assay, rather than actual low IL-17A levels [42]. A robust multiplex GCF study showed a reduction of IL-17A concentrations post-NSPT at a subsequent follow-up, although this measurement appeared later than in the other studies [34]. Overall, the most effective and reliable local marker is IL-17A, which typically appears approximately 6–8 weeks after treatment and stabilises around 3 months, correlating with clinical improvements (reduction in PD, reduction in BOP, and increase in CAL) [15,16,23,24,34,37,39,40].

#### 3.5.2. IL-23

IL-23 exhibited a local decline after NSPT, encouraging clinical outcomes proximal to the treatment time surrounding many of the designs and collections. In pooled chronic/aggressive periodontitis, GCF IL-23 showed a downward trend for >6 weeks [40], and in generalised aggressive periodontitis, GCF IL-23 trended down at >3 months alongside IL-17A [29]. A large multiplex GCF cohort also suggested a post-NSPT reduction in IL-23 levels at a later follow-up [24]. As with IL-17A, unit choice and pre-analytics matter; also, studies reporting just concentrations without dedicated volume consideration are subject to volume-related artifacts, especially in earlier follow-ups [27,28,29,42]. Overall, the trend is a decrease in magnitude and discernibility related to assay platform and sampling procedures [24,34,40].

#### 3.5.3. IL-21

Evidence concerning IL-21 is limited to one cohort, which reported a significant decline in GCF IL-21 approximately 6 weeks following NSPT, coinciding with a reduction in IL-17A; meanwhile, signals associated with Th2 (IL-4, GATA3) increased, while Th1 markers (IFN-γ, T-bet) remained stable [23]. This pattern is biologically plausible for reduced Th17 activity with partial counter-regulation; however, further independent confirmation is necessary.

#### 3.5.4. IL-22

Evidence for IL-22 is limited: GCF IL-22 levels were near the lower measurement range and did not change significantly four weeks after phase I therapy. Accordingly, the factor making evidence certain regarding an IL-22 effect is very low systemic (serum) cytokine kinetics after NSPT.

#### 3.5.5. IL-17A and IL-23

Systemic responses had smaller magnitudes and temporal delay compared to the GCF. Serum IL-17A showed a slight decrease by ~1 month, though this change was not statistically significant [35,37]. Serum IL-23 findings were mixed: decreased levels were found in a generalised aggressive periodontitis cohort at ~3 months in concert with IL-17A [34], and no marked change was seen at ~6 months in a mixed chronic/aggressive cohort [37]. In a 12-month serum/saliva cohort, Th17 axis cytokines in serum were undetectable and/or unable to demonstrate robust directional assignment for serum IL-17A/IL-23 in light of clinical improvement [38]. In summary, these trajectories supported the existence of a relatively modest and delayed systemic dampening phase in relation to the prior local signal, which was stronger and occurred earlier [34,35,37,38].

#### 3.5.6. Counter-Regulation (e.g., IL-17E/IL-25 Ratio)

A more extended follow-up serum study that assessed IL- 17A showed no apparent decline in IL-17A; however, because of a greater increase in IL-17E (IL 25), the IL-17A:IL-17E ratio fell, with a range between ~6 and 25 weeks showing a trend away from Th17 bias despite stable/increased levels of IL-17A [36]. This usage of a ratio-based counter-regulatory readout is mechanistically plausible in mucosal immunology and may also provide complementary pharmacodynamic information regarding absolute IL-17A levels when systemic changes are more subtle [36].

#### 3.5.7. Bone Coupling Mediators (sRANKL/OPG)

Short interval data (≈4 weeks) produced variable early changes: sRANKL was unchanged while OPG was reduced in GCF despite favourable clinical improvement [16]. These observations provide the perspective that bone coupling markers might respond on different timeframes than inflammatory mediators, and that only reporting early concentrations would be misleading without volume capture [15,16]. Mechanistically, it is logical to expect bone coupling shifts with continued inflammatory regulation due to associations between RANKL-mediated osteoclastogenesis and Th17 signalling. However, strong longitudinal evidence is scarce [16,20,43].

#### 3.5.8. Clinical–Biochemical Coupling (PD, CAL, BOP vs. Cytokines)

Data from all cohorts suggest a clinical improvement after NSPT (e.g., PD reduction, BOP reduction, and/or CAL gain). Biochemically, there were also changes (e.g., GCF IL-17A/IL-23 ↓) that occurred concomitantly with these clinical improvements in the earlier time period (≈6–8 weeks with interventions in the local way; ≈3–6 months in the systemic way) [15,23,34,36,37,39,40]. In those longer time frame cohorts, the salivary Th17 axis cytokines (IL-17A/IL-17F/IL-23) also decreased in the more extended time periods of 3–12 months, while blood serum Th17 markers were frequently undetectable [38]. However, pairing (≤4–6 weeks) was less clear because of GCF volume-induced concentration artifacts (concentration ↑ while amount ↔) and variability in assay detection thresholds [15,16]. In addition, given that a lot of GCF outcomes are site-level and within-person and analyses rarely consider clustering, point estimates for effects may be imprecise, emphasising the need for cluster-aware models and standardised pre-analyses [24,28,44] in future longitudinal studies.

Overall, GRADE provides low-certainty evidence that GCF IL-17A decreases after NSPT, with low certainty for serum IL-17A/IL-23 and GCF IL-21, and very low certainty for IL-22 and for sRANKL/OPG. This reflects the updated study-level outcomes: most cohorts showed decreased GCF IL-17A but with methodological limitations that reduce certainty; IL-22 did not change significantly in the lone GCF cohort and was near the assay’s detection range, yielding very-low-certainty; and GCF IL-23 showed a general downward trend across limited, heterogeneous studies (Table 4).

## 4. Discussion

Across diverse cohorts, non-surgical periodontal therapy (NSPT) is generally associated with the attenuation of Th17-axis activity, most clearly in gingival crevicular fluid (GCF). Declines in GCF IL-17A generally emerge by approximately 6–8 weeks and, where measured, show uniform suppression around 3 months [23,24,34,39,40]. Two early-window exceptions qualify this pattern: (i) at 4 weeks, one cohort noted an apparent increase in IL-17A concentration despite no change in total amount, an artefact explained by the post-therapy contraction of GCF volume that inflates concentration-only metrics [16]; and (ii) another cohort reported frequent GCF IL-17A non-detects at baseline and follow-up, underscoring assay sensitivity constraints for this analyte in GCF [15]. A large, GCF study corroborated post-NSPT IL-17A decline and situated it within a broader local program: concurrent decreases in IL-1β, RANTES, and sCD40L, coupled with increases in IP-10 and MMP-9, are compatible with inflammatory resolution and matrix remodelling at treated sites [24].

Systemic responses of these local shifts are smaller and delayed. Serum IL-17A decreased by ~1 month in chronic periodontitis (with/without type 2 diabetes) [35] and by ~6 months in generalized aggressive periodontitis [37]. In contrast, another cohort displayed rises in both IL-17A and IL-17E, yet a declining IL-17A:IL-17E ratio over 6–25 weeks—suggesting a net shift away from Th17 bias despite stable or elevated IL-17A levels [36]. Twelve-month comparisons across saliva and serum reinforce compartment-specific kinetics: salivary Th17 cytokines (IL-17A/IL-17F/IL-23) generally decreased after therapy, whereas serum Th17 markers were often undetectable; more apparent systemic change was seen for IL-1β/IL-6/TNF-α (decreases) and IL-31 (increase) [38]. For IL-23, directionality was consistently downward, but timing varied: decreases were seen in GCF and serum at 3 months in aggressive periodontitis [34], in GCF by ~6 weeks in pooled chronic/aggressive cohorts [40], and again in later follow-up multiplex GCF data [24], while one mixed cohort showed no serum change at 6 months [37]. IL-21 decreased locally at ~6 weeks in the only available study [23], whereas IL-22 showed no significant change at a similar time point in the single GCF cohort that measured it [41]. Early bone coupling readouts were inconsistent (sRANKL unchanged; OPG decreased at 4 weeks) [16].

Taken together, the certainty of evidence is low to very low. There is low-certainty evidence that GCF IL-17A decreases after NSPT; the certainty is also low for serum IL-17A/IL-23 and GCF IL-21, and very-low-certainty for GCF IL-22 and sRANKL/OPG because of single-study coverage, small samples, pre-analytic heterogeneity, and site-specific designs [15,16,23,24,34,35,37,38,39,40,41]. This compartment- and time-resolved synthesis extends prior qualitative and quantitative reviews of GCF cytokines after NSPT [26]. The recurring pattern—early local dampening with slower systemic responses—aligns with mucosal Th17 biology and periodontal pathophysiology [6,7,45,46,47]. Prior meta-analyses emphasized methodological heterogeneity [26]; our synthesis converges on the same variability but adds operational guidance: local responses are most detectable at ~6–8 weeks and systemic changes are most detectable at ~3–6 months, and interpretation should prioritize total amount over concentration when GCF volume is captured [27,28,29,42]. Recent additions—12-month saliva follow-up and a 64-analyte GCF panel—replicate IL-17A/IL-23 decreases post-NSPT [24,38], and other syntheses of the IL-23/IL-17 axis reach similar directional conclusions despite dispersed methods [48,49].

The post-debridement attenuation of Th17 activity is plausible and expected. IL-17A induces neutrophil-recruiting chemokines, G-CSF and RANKL, linking epithelial irritation to osteoclastogenesis and periodontal tissue loss [45,46,50]. The IL-17–RANKL association provides a credible pathway from inflammation to bone resorption [21,22,43,46,47,48,49,50]. By reducing the antigenic burden of a dysbiotic subgingival biofilm, debridement plausibly shifts the local immune set-point away from Th17 dominance, reflected by decreases in IL-21/IL-17A and counter-regulatory increases in IL-4/GATA-3 in the phenotyped cohort [23,46,51]. The IL-17A:IL-17E (IL-25) ratio offers an integrative lens: ratio reduction—even with stable IL-17A—is consistent with an enhanced anti-Th17 milieu [24,47]. Broader panel changes (declining IL-1β, RANTES, sCD40L with rising IP-10 and MMP-9) support a transition toward resolution and remodelling [24]. Salivary Th17 reductions alongside minimal serum detectability reinforces compartment-specific sensitivity [38]. Clinically, any chairside use of pro-inflammatory biomarkers should remain exploratory, only aiding subclinical monitoring alongside established clinical parameters and within research frameworks until validated [52,53,54,55,56].

Th17-axis markers are not yet suitable for routine chairside monitoring due to pre-analytic variability (such as collection methods, elution factors, storage, and freeze–thaw cycles), differences in measurement units, and assay sensitivity. These factors support panel-based research approaches rather than single-analyte decision rules [57]. As benchmarks, established inflammatory biomarkers such as MMP-8 have progressed to point-of-care formats and show reproducible associations with periodontal activity, whereas Th17-axis cytokines remain research-oriented and assay-sensitive [57,58,59]. Likewise, classic mediators (e.g., IL-1β) consistently decline with successful therapy, as observed in multiplex GCF and longitudinal saliva/serum cohorts; any Th17-axis panel would need to demonstrate added value beyond these markers in discrimination, calibration, and net-benefit analyses [24,38].

Any clinical panel should progress through staged evaluation—analytical validity, clinical validity, and clinical utility—before use [60,61]. Future diagnostic studies of Th17-axis biomarkers should adhere to established reporting and appraisal standards for tests, including the STARD reporting checklist and QUADAS-2 risk-of-bias framework, to ensure analytical rigor and clinical validity [62,63].

A well-constructed pragmatic composite could be based on GCF IL-17A (ideally the total quantity with documented volume), IL-23, and relevant counter-regulators such as IL-17E/IL-25. This approach should include predefined thresholds, calibration parameters, discrimination criteria, and appropriate decision-analytic metrics [29]. Emerging multiplex GCF datasets and saliva/serum cohorts support a multi-analyte, multi-compartment framework; site-level signals should be analysed with cluster-aware methods and aligned to PD, CAL, and BOP endpoints [24,34,38,44].

Methodologically, these cohorts share the strengths of prospective pre–post designs with paired sampling and consistent clinical improvement [15,16,23,34,35,36,37,38,40,41]. At the review-process level, a further limitation is that the protocol, although developed a priori, was not registered in a public database (e.g., PROSPERO), potentially increasing the theoretical risk of unreported protocol deviations despite our inclusion of the full protocol and search strategy in the Supplement. The evidence base now includes 12-month saliva and later GCF follow-up as well as multiplex platforms [24,38]. Some limitations include small, single-center samples; the absence of cluster-robust modeling for site-within-person structures; the pooling of phenotypes without sufficient adjustment; notable pre-analytic variability; and reporting based solely on concentration, which can be misleading when GCF volume decreases after therapy [16,25,26,27,29,42]. Imprecision from assay sensitivity and the handling of LOD/non-detects further curtailed inference (including frequent systemic non-detects in one cohort) [15,37,38,41], and inconsistent follow-up schedules plus variable systemic responses precluded quantitative meta-analysis in prior work and in the present synthesis [25,26]. Mixed chronic/aggressive cohorts under pre-2018 definitions introduce residual confounding tied to phenotype-specific Th17 activity [17,64,65,66,67].

Systemic IL17A decreases were more evident in generalized aggressive periodontitis than in chronic periodontitis, whereas GCF IL17A reductions were consistent across phenotypes in the ~6–8-week window [19,34]. Mapping CP/AgP cohorts onto the 2018 staging/grading schema could alter Th17 readouts by shifting risk strata (e.g., Grade C). Within that framework, many of the “chronic periodontitis” cohorts would now cluster around Stage III, with Grades B and C reflecting different risk profiles. Grade C cases typically exhibit hyper-inflammatory phenotypes and more rapid bone loss, which mechanistically would be expected to show higher baseline IL-17/IL-23 activity and potentially slower or less complete normalization after NSPT. Grade B cases may have less extreme Th17 skewing and a more homogeneous decline in IL-17/IL-23 following debridement. Future work should stratify analyses by stage/grade to reduce phenotype-related confounding [17,64].

Future studies should embed (i) explicit volume capture (e.g., Periotron or gravimetry); (ii) predefined elution buffers/volumes; (iii) −80 °C storage with minimal freeze–thaw cycles; (iv) transparent LOD handling; and (v) total amount as the primary GCF metric, with concentration reported secondarily [27,28,29,42]. Protocols should be registered with prespecified assessment windows (~6–8 weeks local; ~3–6 months systemic) and use cluster-robust or mixed-effects models for site-level outcomes. Multicentre trials should evaluate multiplex panels and cross-matrix performance (GCF and saliva) using AUC, calibration, and net-benefit analyses against clinical improvement, with comparative analyses performed by phenotype (CP vs. AgP; staged/graded categories) and by systemic modifiers (e.g., T2DM) to strengthen generalisability [18,35,60].

## 5. Conclusions

Non-surgical periodontal therapy (NSPT) generally associated with reductions Th17-related inflammation—especially IL-17A—in gingival crevicular fluid (GCF), typically within 6–12 weeks, based on small, non-randomized pre–post cohorts. Some early anomalies arise from reduced GCF volume or assay limitations. Broader cytokine profiling confirms IL-17A reduction along with inflammatory resolution markers (e.g., IL-1β, RANTES, sCD40L ↓; IP-10, MMP-9 ↑). Systemic effects are smaller and slower, with mixed IL-17A/IL-17E responses suggesting reduced Th17 bias. IL-23 and IL-21 generally decrease, while IL-22 data are limited. Because concentration-based measures can mislead when fluid volume changes, total cytokine amounts are preferred. Overall evidence certainty is low to very low, consistent with the GRADE assessments, and Th17-axis biomarkers are not yet clinically reliable for routine decision-making.

Clinically, tracking post-NSPT shifts in GCF IL-17A/IL-23 alongside standard indices could help identify non-responders early, tailor recall intervals, and guide adjunctive therapy in higher-risk patients [23,24,60]. Before routine adoption, preanalytical standardization, harmonized reporting (concentration vs. amount/site), and validation against clinical endpoints are required [25,26,27]. Accordingly, Th17-axis markers should be viewed as candidate adjuncts to established measures rather than stand-alone diagnostics at this time [24,25,60].

Given methodological heterogeneity, risk of bias, and assay limitations, these biomarkers should not yet guide individual patient care; rather, they are emerging research tools that may, with standardization and validation, complement clinical indices in the future.

Future work should refine standardized, validated cytokine panels (e.g., IL-17A, IL-23, IL-17E/IL-25) for correlation with clinical outcomes (PD, CAL, BOP).

## Figures and Tables

**Table 1:** Characteristics of included studies.

Study	Design & Sample	Compartment	Analytes	Follow-Up	Clinical Outcome	Main Th17-Relevant Finding
Zhao et al. (2011) [23]	Prospective pre–post; ~30 adults with chronic periodontitis	GCF; peripheral CD4^+^ T cells	IL-17A, IL-21, IL-4, IFN-γ (ELISA); RORC, T-bet, GATA-3 (qPCR); IL-17^+^ CD4^+^ (flow cytometry)	≈6 weeks	↓ PD, ↓ BOP, ↑ CAL	GCF IL-17A ↓, IL-21 ↓, IL-4 ↑; IFN-γ ↔; RORC ↓, GATA-3 ↑; circulating IL-17^+^(incl. IL-17^+^IFN-γ^+^) CD4^+^ cells ↓
Buduneli et al. (2009) [16]	Prospective pre–post; smokers vs. nonsmokers	GCF	IL-17A, sRANKL, OPG (ELISA)	4 weeks	Clinical improvement reported (PD/BOP)	IL-17 concentration ↑ but total amount ↔; sRANKL ↔; OPG ↓; trends not modified by smoking
Cifcibaşı et al. (2015) [34]	Prospective pre–post; generalized aggressive periodontitis	GCF; serum	IL-17, IL-23, MPO (ELISA)	3 months	↓ PD, ↑ CAL	Local and systemic IL-17 ↓ and IL-23 ↓; MPO ↓
Jayakumar et al., (2018) [35]	Prospective pre–post; CP ± T2D	Plasma	IL-17A (ELISA)	1 month	↓ PD, ↑ CAL	Plasma IL-17A ↓ post-NSPT; magnitude may differ by diabetes status
Nile et al. (2016) [36]	Prospective pre–post; chronic periodontitis	Serum	IL-17A, IL-17E/IL-25 (ELISA); IL-17A:IL-17E ratio	6 & 25 weeks	↓ PD, ↑ CAL	Trajectory consistent with reduced Th17 bias over time (ratio shift)
Duarte et al. (2010) [37]	Prospective pre–post; generalized CP/AgP	Serum	IL-17, IL-23, TNF-α, IFN-γ, IL-4 (ELISA)	6 months	↓ PD, ↑ CAL	Serum IL-17 ↓ in AgP; IL-23 ↔; TNF-α ↓ in AgP; IFN-γ, IL-4 ↔.
Medara et al. (2020) [38]	Prospective cohort; 54 periodontitis vs. 40 healthy; baseline, 3, 6, 12 mo during supportive therapy	Serum & saliva (participant-level)	15 Th17-related cytokines (Luminex Th17 panel)	3, 6, 12 months	↓ PD, ↓ BOP vs. baseline	Saliva: IL-17A/IL-17F/IL-23 ↓ with therapy; Serum detection low; Serum IL-31 ↑
Teles et al. (2025) [24]	Longitudinal monitoring; H (n = 112), P (n = 302); site-level progressing vs. stable; bimonthly ×12 mo; post-NSPT 6 mo	GCF (site-level)	64-plex inflammatory panel (Milliplex; Bio-Plex 200)	12 mo natural history + 6 mo post-NSPT	↓ PD, ↓ BOP, ↑ CAL (overall); ↑ sites with PD < 4 mm after NSPT	Monitoring: progressing sites showed higher IL-1β (±IL-17 profile); After NSPT: IL-17A ↓, IL-1β ↓, sCD40L ↓, RANTES ↓; IP-10 ↑
Gür et al. (2022) [39] *	Prospective (RCT context: SRP ± laser)	GCF	IL-17, IL-10, TWEAK, sclerostin (ELISA)	~1 and 3 months (primary window 3 months	↓ PD, ↓ BOP, ↑ CAL	IL-17 ↓; IL-10 ↑; adjunct laser did not obscure NSPT-linked IL-17 ↓
Haghi et al. (2020) [40]	Prospective pre–post; CP and AgP pooled	GCF	IL-17, IL-23 (ELISA)	≈6 weeks	Clinical improvement reported	IL-17 ↓; IL-23 ↓
Pradeep et al. (2009) [15]	Prospective pre–post; health/gingivitis/CP; CP re-sampled post-SRP	GCF	IL-17, IL-18 (ELISA)	6–8 weeks	↓ PD, ↓ BOP	Baseline: IL-17 variably detectable; post-NSPT: IL-18 ↓; IL-17 often undetectable
Nejadi et al. (2018) [41]	Prospective pre–post; chronic periodontitis	GCF	IL-22, S100A12 (ELISA)	≈6 weeks	↓ PD, ↓ BOP	No significant change in GCF IL-22 or S100A12 pre–post NSPT

↓ = decrease after NSPT; ↔ = no measurable change; ↑ = increase. Mixed = different directions across time points; Div. = divergent metrics (e.g., concentration ↑ while total amount ↔); ND = not detected (<LOD); NR = not reported. Arrows indicate changes from baseline at the study’s primary follow-up (≈6–8 weeks for GCF; ≈3–6 months for serum). When both total amount and concentration are reported with explicit GCF volume capture, the direction reflects total amount. * Studies with an adjunct therapy arm. Only the SRP-alone arm (or an arm with no differing cytokine trend from SRP) was included in analysis.

**Table 2:** Compact numeric outcomes after NSPT (pre → post with variability combined; Δ, %Δ, *p*).

Study (Ref)	Disease/Cohort	Specimen	Analyte	Unit	Follow-Up	n	Pre (Mean ± SD or Median [IQR])	Post (Mean ± SD or Median [IQR])	Reported *p*	Direction
Zhao 2011 [23]	CP (pre–post)	GCF	IL-17A	ng/mL	≈6 weeks	30	63.57 ± 19.60	39.91 ± 10.97	<0.05	decrease
Zhao 2011 [23]	CP (pre–post)	GCF	IL-21	ng/mL	≈6 weeks	30	80.34 ± 39.42	31.77 ± 13.26	<0.05	decrease
Buduneli 2009—non-smokers [16]	CP (NS)	GCF	IL-17A (concentration)	pg/mL	4 weeks	10	2.18 ± 1.46	5.87 ± 8.43	NR	increase
Buduneli 2009—non-smokers [16]	CP (NS)	GCF	IL-17A (total amount)	pg/two samples	4 weeks	10	1.41 ± 0.49	1.34 ± 0.37	NR	no significant change
Buduneli 2009—smokers [16]	CP (S)	GCF	IL-17A (concentration)	pg/mL	4 weeks	10	1.57 ± 1.23	3.50 ± 4.10	NR	increase
Buduneli 2009—smokers [16]	CP (S)	GCF	IL-17A (total amount)	pg/two samples	4 weeks	10	1.36 ± 0.42	1.17 ± 0.25	NR	no significant change
Cifcibaşı 2015 [34]	GAgP	Serum	IL-17A	pg/mL	3 months	19	37.56 ± 17.41	27.69 ± 13.86	0.014	decrease
Cifcibaşı 2015 [34]	GAgP	Serum	IL-23	pg/mL	3 months	19	258.63 ± 241.75	168.39 ± 177.75	<0.001	decrease
Cifcibaşı 2015 [34]	GAgP	GCF	IL-17A	pg/mL	3 months	19	2246.92 ± 294.67	1858.58 ± 1425.85	0.013	decrease
Cifcibaşı 2015 [34]	GAgP	GCF	IL-23	pg/mL	3 months	19	675.49 ± 444.02	400.12 ± 241.63	<0.001	decrease
Jayakumar 2018 [35]	CP (non-diabetic)	Plasma	IL-17A	pg/mL	1 month	20	0.18 ± 0.03	0.16 ± 0.02	0.08 (not significant)	no significant change
Jayakumar 2018 [35]	CP + T2DM	Plasma	IL-17A	pg/mL	1 month	20	0.21 ± 0.04	0.16 ± 0.03	0.09 (not significant)	no significant change
Nile 2016 [36]	CP (FM-SRP & Q-SRP)	Serum	IL-17A:IL-17E ratio	unitless	25 weeks	40	NR	NR	<0.05 (ratio lower than baseline)	decrease (ratio)
Duarte 2010 [37]	GAgP	Serum	IL-17A	pg/mL	6 months	14	NR	NR	0.04	decrease
Duarte 2010 [37]	GCP	Serum	IL-23	pg/mL	6 months	14	NR	NR	not significant	no significant change
Medara 2020 [38]	Periodontitis	Serum	IL-17A	pg/mL	3–12 months	54	often below detection	often below detection	NR	not detectable
Medara 2020 [38]	Periodontitis	Saliva	IL-17A	pg/mL	up to 12 months	54	NR (median reported in figure)	NR (median reported in figure)	<0.05 (overall)	decrease
Gür 2022—SRP arm [39]	CP (control)	GCF	IL-17A	ng/L	3 months	20	117.67 ± 10.77	115.19 ± 11.34	0.001 (within-arm)	decrease (small)
Gür 2022—SRP + laser [39]	CP (laser)	GCF	IL-17A	ng/L	3 months	20	117.66 ± 10.25	106.88 ± 7.56	0.001 (within-arm); 0.025 (between-arm at 3 months)	decrease
Haghi 2020 [40]	CP	GCF	IL-17A	pg/mL	~6 weeks	25	1.40 ± 1.36	0.96 ± 0.44	0.048	decrease
Haghi 2020 [40]	CP	GCF	IL-23	pg/mL	~6 weeks	25	80.60 ± 132.10	1.41 ± 3.63	0.001	decrease
Haghi 2020 [40]	AgP	GCF	IL-17A	pg/mL	~6 weeks	29	0.91 ± 0.74	0.51 ± 0.54	0.022	decrease
Haghi 2020 [40]	AgP	GCF	IL-23	pg/mL	~6 weeks	29	32.27 ± 63.59	2.34 ± 4.70	<0.001	decrease
Nejadi 2018 [41]	CP	GCF	IL-22	ng/mL	~4 weeks	22	mean low; exact number not reported	no significant change	not significant	no significant change
Nejadi 2018 [41]	CP	GCF	S100A12	ng/mL	~4 weeks	22	mean low; exact number not reported	no significant change	not significant	no significant change
Pradeep 2009 [15]	CP (pre vs. post)	GCF	IL-18	pg/µL	~6–8 weeks	20	450.54 ± 276.83 (pre-treatment CP)	89.09 ± 66.69 (post-treatment CP)	<0.001	decrease
Pradeep 2009 [15]	CP/healthy/gingivitis	GCF	IL-17A	pg/µL	—	—	approximately zero across groups	approximately zero across groups	—	not detectable
Teles 2025 [24]	Stage II–III periodontitis (site-level)	GCF	IL-17A	pg/mL	6 months post-NSPT	302	model-based; raw means not reported	decrease after NSPT (mixed model)	reported as significant (q < 0.05)	decrease

Δ = post − pre; %Δ = 100 × Δ/Pre. *p* (reported) = author-reported test (within-arm, between-arm, overall, or model); Abbrev: NSPT = non-surgical periodontal therapy; SRP = scaling & root planing; GCF = gingival crevicular fluid; CP = chronic periodontitis; AgP = aggressive periodontitis; GAgP = generalized aggressive periodontitis; LOD = limit of detection; NR = not reported. Units are as reported (pg/mL, ng/mL, ng/L, pg/µL, pg/site); ratios are unitless. GCF note: concentration (e.g., pg/µL) can diverge from amount/site (e.g., pg/site) if crevicular volume changes. Short labels: not reported (both) = no raw pre/post means; undetectable (both) = values < LOD at both timepoints; model-only (no raw means) = effects from a statistical model, no raw means; median (approx) = medians estimated from a figure, Δ/%Δ approximate. Units are presented as reported in the original publications (pg/mL, ng/mL, ng/L, pg/µL), unitless ratios.

**Table 3:** Pre-analytical and analytical reporting by study.

Study	Site-Matching	Device + Dwell	Volume Capture	Elution Buffer	Storage (°C)	Freeze–Thaw Control	Unit Reported	LOD/LOQ Handling	Assay Brand/Model
Zhao 2011 [23]	Yes (same tooth pre/post)	Paper strip (2 × 20 mm), 30 s	Yes (strip weight/volume)	PBS 200 µL	−80 °C	NR	Concentration (ng/mL)	NR (brand LLDs available)	R&D Systems (Quantikine)
Buduneli 2009 [16]	Likely (same 4 sites); not explicit	PerioPaper, 30 s	Yes (Periotron 8000)	PBS 350 µL	−40 °C	NR	Amount & concentration	LLD given (IL-17 0.002 pg/mL); handling NR	ELISA kits (vendor NR)
Cifcibaşı 2015 [34]	Unclear (deepest 4 sites)	PerioPaper, 30 s	No	PBS 350 µL + 0.05% Tween-20; +4 °C overnight	−80 °C	NR	Concentration (GCF & serum)	LLDs reported; handling NR	**Diaclone** (IL-17/IL-23); eBioscience (MPO)
Jayakumar 2018 [35]	N/A (serum)	N/A	N/A	N/A	−80 °C	NR	Concentration (plasma)	NR	DIACLONE (ELISA development kit)
Nile 2016 [36]	N/A (serum)	N/A	N/A	N/A	−80 °C	NR	Concentration (serum)	LLD 1.9 pg/mL (IL-17A/E); handling NR	Peprotech (IL-17A/E); R&D (TNF/IL-6)
Duarte 2010 [37]	N/A (serum)	N/A	N/A	N/A	−80 °C	NR	Concentration (serum)	Below LOD scored 0 pg/mL	ELISA (vendor NR)
Medara 2020 [38]	N/A (serum & saliva)	N/A	N/A	N/A	−80 °C (serum & saliva; processed ≤ 2 h)	NR	Concentration (pg/mL)	Detection frequency noted (serum low); LOD/LOQ handling NR	Bio-Plex Pro Human Th17 Cytokine Panel (Bio-Rad)
Teles 2025 [24]	Yes (site-level; progressing vs. stable; no rescue sites in analyses)	PerioPaper; 30 s; 1–2 mm	No (concentration from elution)	NR (standard Milliplex protocols)	−80 °C (immediate freeze)	NR	Concentration (pg/mL)	Assay performance in Table S3; LOD/LOQ handling NR	Milliplex (MilliporeSigma); read on Bio-Plex 200 (Bio-Rad)
Gür 2022 [35]	Yes (deepest tooth sampled across time)	PerioPaper, 30 s	Yes (Periotron 8000)	PBS 800 µL (sterile)	−20 °C	NR	Concentration (GCF)	LLDs provided; handling NR	Bioassay (IL-17/IL-10/TWEAK); Elabscience (sclerostin)
Haghi 2020 [40]	Unclear (two deepest pockets at each time)	PerioPaper, 30 s	No	PBS 150 µL; 10,000 rpm × 30 min	−20 °C	NR	Concentration (GCF)	NR	Bender MedSystems (IL-17A, IL-23)
Pradeep 2009 [15]	Yes (same site pre/post)	Microcapillary pipettes; ≤10 min cap	Yes (fixed 1 µL/sample)	N/A (direct GCF)	−70 °C	NR	Concentration (pg/µL)	Kit sensitivity ≈15 pg/mL; ND frequently 0	R&D Systems (IL-17); Bender MedSystems (IL-18)
Nejadi 2018 [41]	Yes (same two sites)	PerioPaper, 30 s	No	PBS 50 µL; centrifuge; −70 °C 24 h	−20 °C (then −70 °C 24 h)	NR (≥1 cycle implied)	Concentration (GCF)	NR	eBioscience (IL-22); Biovendor (S100A12)

NR = not reported; N/A = not applicable (serum). “Amount & concentration” implies explicit or inferred GCF volume capture.

**Table 4:** Summary of findings (GRADE) by outcome and compartment.

Outcome	Specimen	Typical Timeframe	Direction After NSPT (Study-Level)	Studies	GRADE Certainty
IL-17A	GCF	6–8 weeks (often persisting by ≈3 months)	Decrease in most studies	Decrease: Zhao 2011 [23]; Cifcibaşı 2015 (GCF) [34]; Haghi 2020 [40]; Gür 2022 [39]; Teles 2025 [24]. No significant change: Buduneli 2009 (amount prioritized) [16]. Indeterminate: Pradeep 2009 (undetectable IL-17A) [15].	Low
IL-23	GCF	≈6–8 weeks	Decrease	Decrease: Haghi 2020 [40]; Cifcibaşı 2015 (GCF) [34]. Indeterminate: Teles 2025 [24] (model-based, no raw pre/post means).	Low
IL-21	GCF	≈6 weeks	Decrease (single study)	Zhao 2011 [23].	Low
IL-22	GCF	≈4–6 weeks	No significant change (single study)	Nejadi 2018 [41].	Very low
IL-17A	Serum/plasma	1–6 months	Decrease or neutral; smaller effects	Decrease: Cifcibaşı 2015 (serum) [34]; Duarte 2010 (AgP subgroup) [37]. No significant change: Jayakumar 2018 (plasma) [35]. Indeterminate: Medara 2020 (often below detection) [38].	Low
IL-23	Serum	3–6 months	Mixed	Decrease: Cifcibaşı 2015 (serum) [34]. No significant change: Duarte 2010 [37].	Low
IL-17A	Saliva	3–12 months	Decrease (single study)	Medara 2020 [38].	Low
IL-17A:IL-17E ratio	Serum	6–25 weeks	Decrease (ratio)	Nile 2016 [36].	Low

GRADE summary of findings for post-treatment changes in Th17-axis cytokines after non-surgical periodontal therapy (NSPT), by compartment. Certainty ratings (high, moderate, low, very low) reflect risk of bias, inconsistency, indirectness, imprecision and publication bias.

## Supplementary Materials

The following supporting information can be downloaded at: https://www.mdpi.com/article/10.3390/jcm14238424/s1.
