# Supplementary material for: Cytokine Changes in Gingival Crevicular Fluid and Serum After Non-Surgical Periodontal Therapy in Patients with Periodontitis: A Systematic Review and Narrative Synthesis
Source: J Clin Med. 2025 Nov 27;14(23):8424. doi: 10.3390/jcm14238424 (PMC12692750; doi:10.3390/jcm14238424)
Supplement: Supplementary file 1 [file jcm-14-08424-s001.zip › jcm-3981877-supplementary.pdf]

Appendix A

Supplementary Table S1 - Keyword search

Pubmed

(periodontitis[tiab] OR "periodontal disease"[tiab] OR periodont*[tiab]) AND ("root planing"[tiab] OR "scaling"[tiab] OR "scaling and root planing"[tiab] OR "subgingival debridement"[tiab] OR "subgingival instrumentation"[tiab] OR "periodontal debridement"[tiab] OR "periodontal therapy"[tiab] OR "periodontal treatment"[tiab] OR "nonsurgical periodontal"[tiab] OR nonsurgical[tiab] OR NSPT[tiab] OR "initial periodontal therapy"[tiab] OR "phase I therapy"[tiab] OR "supportive periodontal therapy"[tiab] OR "periodontal maintenance"[tiab] OR PMPR[tiab]) AND (Th17[tiab] OR "interleukin 17"[tiab] OR IL-17[tiab] OR IL17[tiab] OR "interleukin 21"[tiab] OR IL-21[tiab] OR "interleukin 22"[tiab] OR IL-22[tiab] OR "interleukin 23"[tiab] OR IL-23[tiab] OR RORC[tiab] OR "ROR gamma t"[tiab] OR GATA3[tiab] OR "GATA-3"[tiab] OR RANKL[tiab] OR "TNFSF11"[tiab] OR OPG[tiab] OR osteoprotegerin[tiab] OR cytokine*[tiab] OR biomarker*[tiab] OR "inflammatory mediator*"[tiab])

Embase

('periodontitis'/exp OR periodontitis:ti,ab) AND ('root planing'/exp OR 'dental scaling'/exp OR 'subgingival debridement':ti,ab OR 'nonsurgical periodontal':ti,ab OR nonsurgical:ti,ab OR nspt:ti,ab) AND ('interleukin 17'/exp OR 'interleukin 17':ti,ab OR 'il 17':ti,ab OR il17:ti,ab OR th17:ti,ab OR 'interleukin 21'/exp OR 'interleukin 21':ti,ab OR 'il 21':ti,ab OR 'interleukin 22'/exp OR 'interleukin 22':ti,ab OR 'il 22':ti,ab OR 'interleukin 23'/exp OR 'interleukin 23':ti,ab OR 'il 23':ti,ab OR rorc:ti,ab OR 'gata3':ti,ab OR 'gata-3':ti,ab OR rankl:ti,ab OR opg:ti,ab) AND [humans]/lim AND [english]/lim AND [2000-2025]/py

Cochrane

periodontitis "scaling and root planing" IL-17 OR "interleukin 17" OR Th17 OR IL-23 OR IL-21 OR IL-22 cytokine OR biomarker

Scopus

TITLE-ABS-KEY (periodontitis "scaling and root planing" IL-17 OR "interleukin 17" OR Th17 OR IL-23 OR IL-21 OR IL-22 cytokine OR biomarker)

PRISMA

Records identified from databases: PubMed (n = 1,043), Embase (n = 121), Scopus (n = 16), Cochrane RIS (n = 124); total = 1,304.<br>Duplicates removed before screening: n = 71.<br>Records screened (title/abstract): n = 1,233.<br>Records excluded (title/abstract): n = 1,208.<br>Reports sought for retrieval from databases: n = 25.<br>Additional reports sought via handsearching/citation chasing: n = 4.

Total reports assessed for eligibility: n = 29.
Reports excluded, with reasons: n = 17.
Studies included in qualitative synthesis: n = 12.

Supplementary Table S2

Excluded studies (n = 17)

	**Reason for exclusion**
[1] R. Vernal, N. Dutzan, A. Chaparro, J. Puente, M. A. Valenzuela, and J. Gamonal, "Levels of interleukin-17 in gingival crevicular fluid and in supernatants of cellular cultures of gingival tissue from patients with chronic periodontitis," J. Clin. Periodontol., vol. 32, no. 4, pp. 383–389, 2005, doi: 10.1111/j.1600-051X.2005.00684.x.	Not longitudinal pre/post design (cross-sectional only)
[2] S. R. Lester, J. L. Bain, R. B. Johnson, and F. G. Serio, "Gingival concentrations of interleukin-23 and −17 at healthy sites and at sites of clinical attachment loss," J. Periodontol., vol. 78, no. 8, pp. 1545–1550, 2007.	Not longitudinal pre/post design (cross-sectional only)
[3] A. Beklen, G. Tüter, T. Sorsa, R. Hanemaaijer, I. Virtanen, T. Tervahartiala, et al., "Matrix metalloproteinases, interleukin-1, and tumor necrosis factor-α are regulated by interleukin-17 in periodontitis," J. Dent. Res., vol. 86, no. 4, pp. 347–351, 2007, doi: 10.1177/154405910708600408.	Wrong biospecimen (neither GCF, saliva, nor serum)
[4] M. Adibrad, P. Deyhimi, M. G. Hakemi, P. Behfarnia, M. Shahabuei, and L. Rafiee, "Signs of the presence of Th17 cells in chronic periodontal disease," J. Periodontal Res., vol. 47, no. 4, pp. 525–531, 2012, doi: 10.1111/j.1600-0765.2011.01464.x.	Wrong biospecimen (neither GCF, saliva, nor serum)
[5] M. Mazurek-Mochol, K. Serwin, M. Homa, and J. Banach, "Expression of interleukin-17A and -17B in gingival tissue in patients with periodontitis," J. Clin.	Wrong biospecimen (neither GCF, saliva, nor serum)

Med., vol. 12, no. 14, p. 4614, 2023, doi: 10.3390/jcm12144614.	
[6] F. Javed, A. S. Al-Zawawi, K. S. Allemailem, A. Almatroudi, A. Mehmood, D. D. Divakar, and A. A. Al-Kheraif, "Periodontal conditions and whole salivary IL-17A and -23 levels among young adult Cannabis sativa smokers, heavy cigarette-smokers and non-smokers," Int. J. Environ. Res. Public Health, vol. 17, no. 20, p. 7435, 2020, doi: 10.3390/ijerph17207435.	Not longitudinal pre/post design (cross-sectional only)
[7] A. N. Wankhede, P. V. Dhadse, M. Agrawal, and R. Bhisey, "Interleukin-17 levels in gingival crevicular fluid of aggressive periodontitis and chronic periodontitis patients," J. Indian Soc. Periodontol., vol. 26, no. 6, pp. 552–556, 2022, doi: 10.4103/jisp.jisp_47_21.	Not longitudinal pre/post design (cross-sectional only)
[8] Z. Yetkin Ay, G. Yılmaz, M. Özdem, H. Koçak, R. Sütçü, E. Uskun, et al., "The gingival crevicular fluid levels of interleukin-11 and interleukin-17 in patients with aggressive periodontitis," J. Periodontol., vol. 83, no. 11, pp. 1425–1431, 2012, doi: 10.1902/jop.2012.110585.	Not longitudinal pre/post design (cross-sectional only)
[9] O. G. Shaker and N. A. Ghallab, "Interleukin-17 and interleukin-11 levels in gingival crevicular fluid of patients with aggressive and chronic periodontitis: relation to PCR bacterial detection," Mediators Inflamm., vol. 2012, p. 174764, 2012, doi: 10.1155/2012/174764.	Not longitudinal pre/post design (cross-sectional only)
[10] R. Sadeghi, M. Sattari, F. Dehghan, and S. Akbari, "Interleukin-17 and interleukin-23 levels in gingival crevicular fluid of patients with chronic and aggressive periodontitis," Cent. Eur. J. Immunol., vol. 43, no. 1, pp. 76–80, 2018, doi: 10.5114/ceji.2018.74876.	Not longitudinal pre/post design (cross-sectional only)
[11] C. Giannopoulou, I. Cappuyns, J. Cancela, N. Cionca, and A. Mombelli, "Effect of photodynamic therapy, diode laser, and deep scaling on cytokine and acute-phase protein levels in gingival crevicular fluid of residual	Not NSPT-only (adjunct-only or surgical-only)

periodontal pockets," J. Periodontol., vol. 83, no. 8, pp. 1018–1027, 2012, doi: 10.1902/jop.2011.110281.	
[12] R. Pourabbas, A. Kashefimehr, N. Rahmanpour, Z. Babaloo, A. Kishen, H. C. Tenenbaum, and A. Azarpazhooh, "Effects of photodynamic therapy on clinical and gingival crevicular fluid inflammatory biomarkers in chronic periodontitis: a split-mouth randomized clinical trial," J. Periodontol., vol. 85, no. 9, pp. 1222–1229, 2014, doi: 10.1902/jop.2014.130464.	Not NSPT-only (adjunct-only or surgical-only)
[13] R. Mistry, D. Verma, A. Parakh, P. K. Panda, and B. M. Purohit, "Effect of diode laser used as an adjunct to mechanical periodontal treatment on GCF cytokine levels in patients with chronic periodontitis," J. Lasers Med. Sci., vol. 7, no. 4, pp. 250–255, 2016, doi: 10.15171/jlms.2016.44.	Not NSPT-only (adjunct-only or surgical-only)
[14] V. S. Müller Campanile, C. Giannopoulou, G. Campanile, J. A. Cancela, and A. Mombelli, "Single or repeated antimicrobial photodynamic therapy as adjunct to ultrasonic debridement in residual periodontal pockets: clinical, microbiological, and local biological effects," Lasers Med. Sci., vol. 30, pp. 27–34, 2015, doi: 10.1007/s10103-013-1337-y.	Not NSPT-only (adjunct-only or surgical-only)
[15] E. Darabi, Z. Kadkhoda, and A. Amirzargar, "Comparison of the levels of tumor necrosis factor-α and interleukin-17 in gingival crevicular fluid of patients with peri-implantitis and a control group with healthy implants," Iran. J. Allergy Asthma Immunol., vol. 12, no. 1, pp. 75–80, 2013.	Peri-implant disease (no separate periodontal outcomes)
[16] V. O. Severino, M. Beghini, M. F. de Araújo, R. Grespan, A. L. Capelozza, and C. A. Damante, "Expression of IL-6, IL-10, IL-17 and IL-8 in the peri-implant crevicular fluid (PICF) of patients with peri-implantitis," Arch. Oral Biol., vol. 56, no. 8, pp. 823–828, 2011.	Peri-implant disease (no separate periodontal outcomes)

[17] A. Faramarzi, A. Azarbayejani, H. Y. Esfandiari, and F. Shahroudi, "Evaluation of the efficacy of aloe vera gel after periodontal flap surgery on the serum levels of interleukin-17 cytokine: a randomized double-blind clinical trial," Immunopathol. Persa, vol. 9, no. 1, p. e40, 2023, doi: 10.34172/ipp.2023.40.	Not NSPT-only (adjunct-only or surgical-only)

Supplementary Table S3 (GRADE)

ROBINS-I Evidence profiles for post-NSPT Th17-axis cytokines

Outcome (compartment; primary timeframe)	Studies (n)*	Starting level	Risk of bias	Inconsistency	Indirectness	Imprecision	Publication bias	Direction of effect (summary)	Overall certainty
IL-17A (GCF; ~6–8 wk; clear by ~3 mo)	7	Low (observational)	Downgrade 0–1 (a)	No downgrade (b)	No	No/Minor (c)	Suspected (not testable)	Mostly ↓; early 4-wk conc-only artifact (↑ conc, ↔ amount); one study ND	Low
IL-23 (GCF; ~6–8 wk)	3	Low	Downgrade 0–1 (a)	No/Minor (d)	No	Minor (small n)	Suspected	Mostly ↓ (timing varies; platform-dependent)	Low
IL-21 (GCF; ~6 wk)	1	Low	Downgrade 0–1 (a)	Not applicable (single study)	No	Serious (single study) (e)	Suspected	↓ (co-declines with IL-17A; Th2 markers ↗)	Low (see e)
IL-22 (GCF; ~4–6 wk)	1	Low	Downgrade 1 (a)	Not applicable	Minor (matrix OK; phenotype pooling absent)	Serious (single small cohort; near LOD) (f)	Suspected	↔ / uncertain (levels low; assay sensitivity limits)	Very low
IL-17A (Serum; ~1–6 mo)	5	Low	Downgrade 0–1 (a)	Minor (g)	Minor (systemic readout)	Minor–Moderate (low detectability) (h)	Suspected	Small ↓ or neutral; one cohort shows ↓ IL-17A:IL-17E ratio with stable IL-17A	Low

IL-23 (Serum; ~3–6 mo)	3	Low	Downgrade 0–1 (a)	Minor (mixed ↓/↔)	Minor	Moderate (small n; detection limits)	Suspected	Mixed (↓ in AgP at ~3 mo; ↔ in mixed cohorts)	Low

GCF IL-17A: Zhao 2011; Buduneli 2009; Cifcibasi 2015; Haghi 2020; Gür 2022 (SRP arm); Teles 2024; Pradeep 2009.
GCF IL-23: Cifcibasi 2015; Haghi 2020; Teles 2024.
GCF IL-21: Zhao 2011.
GCF IL-22: Nejadi 2018.
Serum IL-17A: Jayakumar 2018; Duarte 2010; Cifcibasi 2015; Medara 2020.
Serum IL-23: Cifcibasi 2015; Duarte 2010; Medara 2020.

Footnotes
(a) Non-randomized pre/post designs; site-within-person clustering; pre-analytical heterogeneity in GCF (device/dwell, elution, storage), and variable volume capture (concentration-only vs amount) elevate ROBINS-I domains.
(b) Direction largely concordant for GCF IL-17A; exceptions are explained by volume contraction artifacts (4-wk conc-only ↑ with amount ↔) and assay non-detects in one older cohort; neither implies true biological discordance.
(c) Cohorts are small but multiple, and one large multiplex GCF dataset corroborates the post-NSPT decline; we did not penalize further beyond starting at Low.
(d) IL-23 direction is consistently downward locally; variation in timing and platform sensitivity explains modest heterogeneity.
(e) Single-study evidence (IL-21) → serious imprecision; retained Low (not "Very low") because the signal is mechanistically coherent and internally controlled in that cohort.
(f) Single small IL-22 cohort with near-LOD values and incomplete volume normalization; overall Very low certainty.
(g) Systemic changes are smaller/delayed vs GCF; one cohort shows a reduced IL-17A:IL-17E ratio despite stable IL-17A, suggesting counter-regulation rather than inconsistency.
(h) Low detectability in serum multiplex (several Th17 cytokines below LOD) limits precision.

Supplementary Table S4 -ROB

**Study (Year)**	**Confounding**	**Selection of Participants**	**Classification of Interventions**	**Deviations from Intended Interventions)**	**Missing Data**	**Measurement of Outcomes)**	**Selection of Reported Result**	**Overall Risk of Bias**
Zhao et al. (2011)	Moderate. No control group – pre/post design. No adjustment for confounders (e.g. disease severity), but all patients had chronic periodontitis and were treated uniformly.	Low. Included consecutive chronic periodontitis patients meeting criteria; no indication of biased selection. Baseline characteristics were reasonably homogeneous (all chronic cases).	Low. All patients received the same non-surgical periodontal therapy (scaling/root planing); intervention classification was clear and consistent	Low. No deviations from intended therapy reported. All participants received standard SRP; no adjunctive treatments or protocol violations noted.	Low. Complete follow-up at 6 weeks for all 30 patients No drop-outs or missing GCF/clinical measurements reported.	Low. Outcomes measured with objective assays. GCF volume was measured (strips weighed and converted to μL) to avoid dilution artifacts Lab analyses (ELISA, PCR, flow cytometry) were standard; although examiners weren't explicitly blinded, results (cytokine levels) are laboratory-derived.	Low. All specified outcomes (IL-17, IL-21, IFN-γ, IL-4 in GCF; Th-cell transcription factors; Th17 cell frequencies) were reported. Both significant and non-significant findings (e.g. IFN-γ no change) were presented, suggesting no selective outcome reporting.	Moderate. No major biases beyond the inherent lack of a control group. The pre-post design and lack of confounder adjustment warrant a moderate overall risk.
Buduneli et al. (2009)	Moderate. No untreated control group; before-vs-after comparisons could be influenced by secular healing. However, the study stratified by smoking status to control that confounder. Remaining	Low. Recruited systemically healthy adults with chronic periodontitis (10 smokers, 10 non-smokers) seeking care. Selection was likely consecutive and not based on prognosis or outcomes. Groups were similar aside from smoking.	Low. Intervention (initial SRP and oral hygiene instruction) was applied uniformly to all patients. No issues in classifying who got the therapy – all subjects received the same treatment.	Low. No deviations reported. All patients adhered to the assigned therapy (single-course SRP) and follow-up at 4 weeks. No co-interventions or protocol deviations noted.	Low. All 20 patients were followed through 4-week sampling; no attrition reported (short follow-up facilitated complete data).	Low. GCF IL-17, OPG, and sRANKL were measured by ELISA with standardized methods. Importantly, GCF volume and total amount vs. concentration were measured and reported,	Moderate. *Selective reporting risk:* Multiple inflammatory mediators were assayed (IL-17, sRANKL, OPG). While all three were reported, emphasis on certain results could introduce bias. The	Moderate. Despite robust outcome measurements (volume captured) and stratification by smoking, the lack of a control and potential selective emphasis of results yield a

	prognostic factors (age, oral hygiene) were not adjusted, so some confounding may persist.					minimizing measurement bias from post-therapy volume reduction. Laboratory blinding not stated, but biochemical outcomes are objective.	authors highlighted the IL-17 concentration increase but did not explicitly comment on IL-17 total amount in text (which remained unchanged). This raises some concern that results for non-significant metrics were downplayed.	moderate overall risk.
Cifcibaşı et al. (2015)	Moderate. No randomized control; comparisons are pre/post in aggressive periodontitis patients. Potential confounders (e.g. disease severity, age) were not adjusted, though all subjects had generalized aggressive periodontitis. A separate healthy control group was included for baseline comparisons, but this does not control	Low. Enrolled 19 systemically healthy patients with generalized aggressive periodontitis and 22 healthy controls. Inclusion/exclusion criteria were clearly defined (no recent antibiotics, non-smokers). No evidence of bias in how patients were chosen or analyzed (cases vs. controls were matched in age range ~28–29 years).	Low. Intervention classification was straightforward: all aggressive periodontitis patients received non-surgical therapy (SRP) and were reassessed 3 months later. No issues of misclassification between treated vs. untreated – each patient served as their own control over time.	Low. Adherence was good – all patients received initial therapy (scaling in 2 visits) and returned at 3 months. No protocol deviations or additional interventions reported.	Low. Complete follow-up: all 19 patients' serum and GCF samples were analyzed at baseline and 3 months. No attrition described.	Moderate. *Measurement bias:* GCF cytokine levels (IL-17, IL-23, MPO) were determined by ELISA. GCF from four pockets was pooled per patient, and results expressed both per mL and per site. However, GCF volume was not individually measured – values are averages per site, potentially obscuring volume changes.	Moderate. Some outcome data reporting was incomplete: variability measures for certain comparisons were not fully reported (e.g. figures given without SD/error bars for some outcomes). This "incomplete variance reporting" suggests a slight risk of selective reporting – e.g. focusing on significant drops in cytokines and	Moderate. The study lacks a concurrent control and had minor reporting and measurement limitations. Overall judged moderate risk of bias.

	longitudinal confounding.					Laboratory blinding was not mentioned. These pre-analytic omissions introduce some uncertainty in the accuracy of GCF measurements	not detailing all variability. Nonetheless, all primary outcomes (IL-17, IL-23, MPO in both compartments) were reported with p-values.	
Gür et al. (2022)	Moderate. Although conducted within an RCT (SRP + laser vs. SRP alone), for this review's purposes each arm can be seen as a cohort pre/post SRP. There is no untreated group, so time-related confounding (e.g. natural healing) is uncontrolled. Randomization in the parent trial did minimize baseline differences between groups, so prognostic confounders within each arm are likely balanced (low risk in that	Low. Participants were those enrolled in an RCT with clear inclusion criteria (chronic periodontitis, systemically healthy). Random allocation to SRP ± laser means no selection bias in group assignment. All SRP-only patients were included in analysis; selection into the study was not tied to outcomes.	Low. Interventions were well-defined and protocolized (standard SRP in all, plus laser in the other arm). No misclassification – treatment delivery followed the assigned protocol ("SRP protocolized") and biomarker sampling was uniform.	Low. No deviations reported. As an RCT, adherence was likely monitored; all patients in the SRP arm received the intended therapy with no cross-overs. Short follow-up (6 weeks or 3 months) was by design and applied to all equally.	Low. Follow-up was complete at the study's endpoint (~6–12 weeks; exact follow-up "short"). The analysis included all randomized subjects, with no losses reported in the biomarker outcomes.	Low. Outcome measurement was robust. Biomarker lab methods were "adequate" – IL-17, IL-10, TWEAK, sclerostin in GCF were measured via ELISA with proper techniques. Likely single-blind: lab assays produce objective readings, reducing detection bias. Volume of GCF may not have been explicitly stated, but any volume artifact would equally affect both arms.	Low. All outcomes specified (the four GCF biomarkers at baseline and follow-up) were reported for both groups. No evidence of selective reporting – positive and null results were presumably presented (the context suggests IL-17 and others were fully reported).	Moderate. Overall moderate risk. Within an RCT design, many biases were low; however, without a non-treatment control and given the short follow-up, a conservative moderate rating is assigned.

	context). Overall moderate, due to absence of a no-treatment control.							
Nejadi et al. (2018)	Serious. No control group and limited adjustment for prognostic factors. All 22 patients had chronic periodontitis, but variability in disease severity or systemic conditions (none reported) could affect IL-22/S100 levels. The study did not adjust or stratify by any such factors. Thus, we cannot be confident that observed (non-)changes are due solely to the intervention rather than patient heterogeneity or secular trends.	Low. A convenience sample of 22 moderate-to-severe periodontitis patients was included. Inclusion/exclusion criteria (no recent antibiotics, etc.) were applied; no evidence that participants were selected based on outcome likelihood. The cohort is small, but that affects precision more than selection bias.	Low. All patients underwent Phase I therapy (scaling and oral hygiene) identically. There was no comparison of different interventions – classification was straightforward (everyone received NSPT).	Low. No deviations reported. All subjects completed the intended treatment and returned ~4 weeks post-therapy for GCF sampling. There were no co-interventions (e.g. no mention of adjunctive drugs) that would bias outcomes.	Low. All 22 patients provided pre- and post-treatment GCF samples; no drop-outs in this short-term study (4-week follow-up). Thus, no concerns about missing outcome data.	Moderate. *Measurement bias:* GCF IL-22 and S100A12 were measured by ELISA, but pre-analytic reporting was incomplete. GCF was collected on strips and eluted in buffer, yet it's unclear if GCF volume was quantified or if assay sensitivity was sufficient (IL-22 levels were very low). Partial methodological details (e.g. 50 µL PBS used for elution) were given, but lack of volume normalization means results are in concentration terms, potentially affected by post-therapy volume reductions. These factors introduce	Low. The study reported both outcomes of interest (IL-22 and S100) before and after NSPT, even though neither showed significant change. Non-significant findings were not withheld – instead the authors focused on correlations and noted the absence of significant changes. No additional outcomes were measured, so selective reporting is not a concern.	Serious. Overall deemed serious risk of bias. The combination of no confounder control in a small cohort and some methodological opacity in measurements lowers confidence in linking NSPT to the observed IL-22/S100 results.

						moderate uncertainty in outcome measurement.		
Jayakumar et al. (2018)	Serious. Potential confounding by systemic condition. This study included two patient groups – chronic periodontitis with and without Type II diabetes – and evaluated the effect of treatment on serum IL-17 in each. Diabetes is a major prognostic factor for inflammation. Although the groups were analyzed separately, any combined interpretation could be confounded by their metabolic differences. No adjustments beyond stratification were made (e.g. no matching for age or periodontal	Low. Selection was based on clear criteria (20 chronic periodontitis patients with well-controlled T2DM, and 20 without diabetes). Patients were of similar periodontal status at baseline (both groups had chronic periodontitis) and were recruited consecutively, not based on outcomes. No evidence of self-selection or differential inclusion that would bias results.	Low. Intervention (initial SRP) was consistently applied to all patients in both groups. There is no confusion in intervention classification – each patient received the same therapy, and groups differ only by diabetic status, not by intervention.	Low. No deviations reported. All patients in both groups completed Phase I therapy and returned at 1 month. There were no co-interventions aside from standard diabetes care (which was stable throughout, as all diabetics were “well-controlled”).	Low. All 40 enrolled patients (20 + 20) were accounted for at 1 month; no drop-outs or missing blood samples are reported. Short-term follow-up virtually guarantees complete data.	Moderate. *Measurement bias:* Outcome = plasma IL-17A, measured by ELISA. Short systemic follow-up (1 month) may be insufficient for full cytokine normalization, possibly underestimating changes (timing issue). Moreover, IL-17 levels in plasma were extremely low (~0.2 pg/mL), pushing the assay’s detection limits. The authors noted that GCF might be a more sensitive medium for IL-17. Lab personnel blinding wasn’t mentioned; however, the assay is objective. Overall, some moderate	Moderate. The study focused on a single outcome (IL-17) and reported it for both groups at baseline and follow-up. Baseline IL-17 values and changes were given (including that diabetics had higher IL-17 at baseline). There was *incomplete variance reporting* – e.g. baseline means ±SD were reported, but post-treatment IL-17 values were described only narratively. Still, all results (including the lack of a statistically significant between-group difference in IL-17 reduction) were conveyed, so outright selective	Serious. Overall high risk, driven primarily by the confounding influence of diabetes. The combination of heterogeneous patient groups and very short follow-up yields serious overall bias concerns, despite otherwise reliable measurements.

	severity), so bias due to diabetes status and related factors is a concern.					concern that the small magnitude of change could be obscured by measurement limitations.	omission is not evident.	
Nile et al. (2016)	Moderate. Uncontrolled before/after design in a periodontal treatment study (likely chronic periodontitis). No parallel control group, so improvements in serum cytokines over ~6 months might partly reflect unrelated changes. However, the longer follow-up (up to 25 weeks) provides a more sustained observation of trends. Key confounders (e.g. systemic conditions, concurrent meds) were not mentioned, presumably controlled via exclusion. Moderate risk due to absence of a comparator and unadjusted	Low. Inclusion criteria were adults with periodontitis (generalized) meeting specific conditions; no biases in selection noted. Sample size was modest (likely ~30, not stated here), but participants were not selected based on outcome.	Low. All patients received non-surgical therapy; no intervention misclassification. In this single-arm study, classification is straightforward.	Low. No deviations reported post-intervention. Subjects underwent standard SRP and maintenance; compliance was not flagged as an issue over the 6–25 week follow-up.	Low. Follow-up was relatively long (6 months), but no attrition was reported. It appears all enrolled subjects completed the study, as outcomes were measured for the whole cohort.	Moderate. *Measurement bias:* Outcome of interest was serum IL-17A and IL-17E (IL-25) levels and their ratio. The authors introduced a ratio outcome (IL-17A:IL-17E) to assess the balance of pro- vs. anti-inflammatory IL-17 family cytokines. While mechanistically interesting, this complicates comparability with other studies and could be seen as an analytical choice made after seeing individual results (IL-17A alone showed no clear decline, IL-17E increased). The lab measurements themselves	Low. Both cytokines (IL-17A and IL-17E) were reported individually and the ratio presented. There is no sign of selective non-reporting – the null finding for IL-17A was discussed alongside the significant rise in IL-17E. The ratio was used to summarize these changes, not to hide them. Thus, outcome reporting appears complete.	Moderate. Overall moderate risk. The lack of a control and the unconventional outcome metric introduce some uncertainty, but all outcomes were measured and reported thoroughly.

	prognostic factors.					(ELISA for IL-17A and IL-17E) were standard, but the emphasis on a derived metric adds moderate risk of interpretation bias.		
Haghi et al. (2020)	Serious. This study pooled two different periodontitis phenotypes (chronic and aggressive) in one trial. Combining generalized aggressive and chronic periodontitis patients can introduce confounding – these groups differ in age and immune response, which may influence cytokine levels. They did stratify analyses by disease type to some extent (reported results for each group), but the interpretation of overall effect is still muddied by	Moderate. 54 patients (mix of aggressive and chronic cases) were selected for GCF sampling. It's unclear if groups were balanced (e.g. aggressive cases younger). The recruitment might have been convenience-based across two phenotypes, which could bias comparisons if, say, more severe cases were in one category. Within each phenotype group, selection was likely unbiased. Given the phenotype imbalance, we assign moderate risk here.	Low. Intervention classification was consistent: all patients underwent initial non-surgical therapy (debridement) with the same protocol. No issues differentiating interventions – every subject received NSPT.	Low. Deviations from intended intervention were not noted. All patients completed SRP and were re-evaluated 6 weeks later. No adjunct therapies or non-compliance events reported.	Low. Follow-up at 6 weeks was completed for all participants; no missing GCF samples or drop-outs mentioned.	Moderate. *Measurement bias:* GCF IL-17 and IL-23 were measured by ELISA. Limited details on GCF volume capture were provided – they collected GCF from two deep sites per patient and eluted in buffer, but did not quantify actual GCF volume. Results were reported in concentration (pg/mL) per 150 µL eluent. Without volume normalization, early post-therapy reductions in GCF volume could falsely elevate concentrations. The short	Low. The study reported both cytokines in both patient groups, before and after therapy. No outcomes were omitted. The data showed significant IL-17 and IL-23 decreases in each phenotype group. There is no suggestion of selective reporting – even the cross-group correlation findings (present in chronic, not in aggressive) were fully described.	Serious. Overall serious risk. The conflation of chronic and aggressive cases (with no control group) is a major limitation, compounded by the lack of GCF volume control in measurements.

	heterogeneity. No control group was included, and no adjustments for other confounders were made. Thus, confounding risk is serious.					interval (6 weeks) magnifies this concern. Lab personnel blinding wasn't stated; however, assays were objective and identical kits used for all. Overall, moderate detection bias risk due to possible volume artifact.		
Duarte et al. (2010)	Serious. A non-randomized "before/after" study comparing two distinct groups – generalized aggressive vs. chronic periodontitis – adds substantial confounding concerns. Baseline differences between GAgP and GCP were evident (aggressive cases had higher systemic IL-17 and TNFα). While each group's pre/post change was	Low. 28 patients with periodontitis (14 GAgP, 14 GCP) and 14 healthy controls were included. Selection into groups was based on periodontal diagnosis, not likelihood of response. All were systemically healthy (aside from periodontal status). There is no indication of bias in who was chosen; groups were similar in demographics except disease type (GAgP patients were younger on	Low. Interventions were clearly classified: all periodontitis patients received non-surgical therapy after baseline sampling. Controls received no intervention (just baseline sampling). There's no misclassification – each subject's status (Aggressive, Chronic, or Healthy) and treatment (yes/no) was correctly assigned.	Low. No deviations reported. Both periodontitis groups underwent SRP and were re-evaluated at 6 months. Adherence was good; no cross-over (healthy controls remained untreated, as appropriate).	Low. Follow-up at 6 months was completed for all 28 treated patients. The study is described as a pilot, but no attrition was noted – presumably all patients in GAgP and GCP groups provided post-treatment serum samples.	Moderate. *Measurement bias:* A broad panel of serum cytokines was measured (TNFα, IFNγ, IL-4, IL-17, IL-23) Laboratory blinding isn't mentioned, but assays (ELISA) are objective. The main bias risk lies in multiple comparisons: with five cytokines and two timepoints, the study might capitalize on chance findings (only TNFα and IL-17 in GAgP	Moderate. The authors reported results for all measured cytokines. Notably, they explicitly state that IL-23 and IFNγ did *not* change after therapy, and IL-4 was mostly undetectable. However, the focus in conclusions is on the significant findings (TNFα, IL-17). This emphasis is expected but does raise a slight concern that the	Serious. Overall high risk of bias. The combination of non-comparable patient groups (GAgP vs. GCP) and multiple outcomes yields a serious risk, despite the thorough reporting of results. A larger, controlled study would be needed to confirm these findings without bias.

	evaluated separately, any general conclusions are complicated by these population differences. No adjustments were made for age or other factors correlated with aggressive vs. chronic periodontitis. Additionally, without a healthy or untreated periodontitis control for the *effect* of therapy, we cannot separate treatment impact from background changes. Thus confounding is rated serious.	average, inherent to the condition).				showed significant drops). Indeed, IL-23 and IL-4 showed no change. The absence of GCF data (this study measured only blood markers) avoids volume artifacts. Overall moderate detection bias – lab methods were fine, but measuring many markers increases the opportunity for measurement error or spuriously significant results.	multiple outcomes could lead to selective emphasis. Since the paper does present the null results in text/figures, we judge reporting bias as moderate, not severe.	
Pradeep et al. (2009)	Moderate. No randomized control – comparisons rely on baseline vs. 6-week post-therapy in the periodontitis group. Confounding by other factors is partly mitigated	Low. Sixty subjects were divided into groups: healthy, gingivitis, chronic periodontitis, and the periodontitis group after treatment (same individuals). Care was taken to age- and gender-match groups, minimizing	Low. Intervention classification is clear: Group 3 patients had SRP (initial therapy), group 4 is simply group 3 post-treatment. Healthy and gingivitis groups received no intervention. There is no ambiguity in who got treated vs. not.	Low. No deviations. All 20 periodontitis patients received scaling and re-evaluation at ~6 weeks. No additional treatments or non-compliance issues reported.	Low. Complete follow-up. Group 3 (20 periodontitis patients) yielded Group 4 (the same 20 after therapy). No patients were lost; all	Serious. *Measurement bias:* The study measured IL-17 and IL-18 in GCF. Detectability issues with IL-17 were significant – IL-17 levels were "nearly zero" in all	Low. Outcomes were reported for all groups. The absence of IL-17 in GCF was explicitly stated – the authors did not hide this null result (indeed it was a primary finding). IL-18	Serious. Overall serious risk. The inability to detect IL-17 (one of two target outcomes) undermines confidence in the findings – this, combined

	by the study design: the same patients were measured before/after, and healthy and gingivitis groups served as baseline references. Still, without a non-treated periodontitis group, we cannot fully exclude natural disease fluctuation. All subjects were Indian population, systemically healthy, matched for age/gender to reduce confounding. Moderate risk due to lack of a parallel control for the treatment effect.	selection differences. Participants were included based on clear clinical criteria (GI, PPD, CAL, bone loss severity), not on outcomes. No attrition between baseline and follow-up for the chronic periodontitis group.		Home care improvements were part of treatment, affecting all similarly.	planned GCF samples were obtained.	groups' GCF, likely due to assay sensitivity (~15 pg/mL) being too low. Essentially, IL-17 was *undetectable* (below the kit's LOD) in most samples, yet the authors had to conclude it's absent rather than potentially present at low levels. This raises a serious risk that a true treatment effect on IL-17 could not be observed due to measurement limitations. IL-18 was measurable and did show expected patterns. Additionally, a unit/reporting inconsistency: IL-17 was reported in pg/µL (given as "0 pg/µL"), whereas IL-18 was detected and reported in pg/sample, but the presentation was somewhat	results (which were positive) were fully reported. No selective non-reporting; the conclusion that IL-17 "cannot be considered a biomarker" was based on the data, albeit the data's reliability is in question.	with the pre-post design without a concurrent control, warrants a serious risk of bias rating.

						confusing (they treated post-treatment as a separate "group 4" rather than paired data). These factors reflect a high risk of measurement error/bias for IL-17.		
Teles et al. (2025)	Serious. Prospective, non-randomized within-subject comparisons after a 12-month monitoring phase without therapy, followed by pre/post assessments 6 months after NSPT. Even with mixed-effects modelling, time-varying behaviours (oral hygiene, plaque control) and patient-level heterogeneity can confound both progression and treatment effects. Rescue criteria were prespecified, but the analyte analyses	Low. Large, multi-centre cohort with clear inclusion/exclusion; systemically healthy, non-smokers, non-diabetics among completers; stable vs progressing sites defined a priori from longitudinal CAL models; no evidence of prognostic selection.	Low. Intervention status/timing were protocolized: 1-year "no-therapy" monitoring then standardized NSPT, with explicit rescue rules. Site status (progression/stability) and treatment windows were prespecified.	Low. Monitoring and post-monitoring NSPT delivered per protocol; included sites were not subjected to rescue; no differential co-interventions reported.	Moderate. Attrition from baseline enrolment to completion; longitudinal, site-level pairing requires availability across visits. Mixed models mitigate imbalance but non-random loss could bias estimates.	Moderate. 64-plex bead-based assays on 30-s Periopaper strips with FDR control; results reported as concentrations (pg/mL). Lack of explicit GCF volume normalization can confound post-NSPT concentration changes due to reduced GCF volume; laboratory assays otherwise robust.	Low. Prespecified multiplex panel reported across time (monitoring and post-NSPT), with both significant and null findings and multiple-testing control; no evidence of selective omission.	Serious

	excluded rescued sites, which does not eliminate confounding in the remaining sites							
Medara et al. (2020)	Moderate. Observational cohort with repeated measures (periodontitis) and matched healthy controls; linear mixed models adjusted for timepoint, age, sex, explored smoking interactions and periodontal parameters. Residual confounding remains without randomization.	Low. Clear inclusion/exclusion (≥16 teeth; PD criteria; systemically healthy; no recent antibiotics), age/sex-matched controls; clinic-based recruitment; no signs of prognosis-based selection.	Low. Periodontitis participants received standard supportive periodontal therapy (debridement every 3 months) at defined intervals; classification was uniform.	Low. Supportive therapy delivered per protocol; no adjuncts likely to differentially affect cytokines were reported.	Moderate. Attrition from 54 periodontitis subjects at baseline to 37 at 12 months; LMMs accommodate unbalanced data, but loss to follow-up could bias effects if not missing at random.	Moderate. Multiplex (Luminex) of 15 Th17-related cytokines in serum and saliva. Serum detection was poor for several Th17 cytokines (e.g., IL-17A/F detected rarely; IL-4/21/22/23 below detection), limiting precision and hampering robust directionality for serum changes; saliva was more informative.	Low. All targeted cytokines reported in both matrices with significant and null results; transparent correlation and predictor analyses; no evidence of selective reporting.	Moderate

Supplementary Table S5 (GCF). GCF volume capture in included studies

Study (year)	Compartment(s)	GCF volume measured?	Method
Zhao et al. (2011)	GCF, peripheral blood	Yes	Strips weighed and converted to μL (strip weight/volume).
Buduneli et al. (2009)	GCF	Yes	Periotron 8000 (electronic GCF volume meter).
Cifcibaşı et al. (2015)	GCF, serum	No	GCF collected on PerioPaper and eluted; no explicit volume quantification.
Jayakumar et al. (2018)	Plasma	Not applicable	Serum/plasma only; no GCF sampling.
Nile et al. (2016)	Serum	Not applicable	Serum only; no GCF sampling.
Duarte et al. (2010)	Serum	Not applicable	Serum only; no GCF sampling.
Medara et al. (2020)	Serum, saliva	Not applicable	Serum/saliva only; no GCF sampling.
Teles et al. (2025)	GCF	No	PerioPaper with fixed elution volume; concentration reported, no volume capture.
Gür et al. (2022)	GCF	Yes	Periotron 8000; deepest tooth sampled across time.
Haghi et al. (2020)	GCF	No	PerioPaper eluted into buffer; no explicit volume measurement.

Pradeep et al. (2009)	GCF	Yes	Mcrocapillary volume per sample.
Nejadi et al. (2018)	GCF	No	PerioPaper eluted; no explicit volume capture.

Supplementary Table S6 – PRISMA Checklist

**Section and Topic**	**Item #**	**Checklist item**	**Location where item is reported**
**TITLE**			
**Title**	1	Identify the report as a systematic review.	Page 1, Line 3
**ABSTRACT**			
**Abstract**	2	See the PRISMA 2020 for Abstracts checklist.	Page 1
**INTRODUCTION**			
**Rationale**	3	Describe the rationale for the review in the context of existing knowledge.	Page 2, Lines 63–75
**Objectives**	4	Provide an explicit statement of the objective(s) or question(s) the review addresses.	Page 2, Lines 69–75
**METHODS**			

**Eligibility criteria**	5	Specify the inclusion and exclusion criteria for the review and how studies were grouped for the syntheses.	Page 2, Lines 78–82
**Information sources**	6	Specify all databases, registers, websites, organisations, reference lists and other sources searched or consulted to identify studies. Specify the date when each source was last searched or consulted.	Page 3, Lines 101–113
**Search strategy**	7	Present the full search strategies for all databases, registers and websites, including any filters and limits used.	Page 3, Lines 107–110; Supplementary 1 (search strings)
**Selection process**	8	Specify the methods used to decide whether a study met the inclusion criteria of the review, including how many reviewers screened each record and each report retrieved, whether they worked independently, and if applicable, details of automation tools used in the process.	Page 3, Lines 102–113
**Data collection process**	9	Specify the methods used to collect data from reports, including how many reviewers collected data from each report, whether they worked independently, any processes for obtaining or confirming data from study investigators, and if applicable, details of automation tools used in the process.	Pages 3–4, Lines 110–137
**Data items**	10a	List and define all outcomes for which data were sought. Specify whether all results that were compatible with each outcome domain in each study were sought (e.g. for all measures, time points, analyses), and if not, the methods used to decide which results to collect.	Page 3, Lines 96–100
**Data items**	10b	List and define all other variables for which data were sought (e.g. participant and intervention characteristics, funding sources).	Page 3, Lines 98–100; Page 4, Lines 129–137

		Describe any assumptions made about any missing or unclear information.	
**Study risk of bias assessment**	11	Specify the methods used to assess risk of bias in the included studies, including details of the tool(s) used, how many reviewers assessed each study and whether they worked independently, and if applicable, details of automation tools used in the process.	Page 4, Lines 142–149; Supplementary Table SROB
**Effect measures**	12	Specify for each outcome the effect measure(s) (e.g. risk ratio, mean difference) used in the synthesis or presentation of results.	Page 4, Lines 147–166
**Synthesis methods**			
**Synthesis methods**	13a	Describe the processes used to decide which studies were eligible for each synthesis (e.g. tabulating the study intervention characteristics and comparing against the planned groups for each synthesis (item #5)).	Page 4, Lines 147–164
**Synthesis methods**	13b	Describe any methods required to prepare the data for presentation or synthesis, such as handling of missing summary statistics, or data conversions.	Page 4, Lines 147–164
**Synthesis methods**	13c	Describe any methods used to tabulate or visually display results of individual studies and syntheses.	Page 4, Lines 147–164; Tables 1–2
**Synthesis methods**	13d	Describe any methods used to synthesize results and provide a rationale for the choice(s). If meta-analysis was performed, describe the model(s), method(s) to identify the presence and extent of statistical heterogeneity, and software package(s) used.	Page 4, Lines 147–164 (qualitative direction-of-effect synthesis; no meta-analysis performed)

**Synthesis methods**	13e	Describe any methods used to explore possible causes of heterogeneity among study results (e.g. subgroup analysis, meta-regression).	Not applicable – no meta-analysis or formal heterogeneity analyses were conducted.
**Synthesis methods**	13f	Describe any sensitivity analyses conducted to assess robustness of the synthesized results.	Not applicable – no formal sensitivity analyses were conducted.
**Reporting bias assessment**	14	Describe any methods used to assess risk of bias due to missing results in a synthesis (arising from reporting biases).	No formal method applied; potential publication bias considered qualitatively within GRADE (Supplementary Table S-GRADE).
**Certainty assessment**	15	Describe any methods used to assess certainty (or confidence) in the body of evidence for an outcome.	Page 4, Lines 162–165 (GRADE methods); Supplementary Table S-GRADE
**RESULTS**			
**Study selection**	16a	Describe the results of the search and selection process, from the number of records identified in the search to the number of studies included in the review, ideally using a flow diagram.	Page 5, Lines 170–177; Figure S1
**Study selection**	16b	Cite studies that might appear to meet the inclusion criteria, but which were excluded, and explain why they were excluded.	Page 5, Lines 173–175; Figure S1; Supplementary 2 (Excluded studies)
**Study characteristics**	17	Cite each included study and present its characteristics.	Table 1, Pages 7–9
**Risk of bias in studies**	18	Present assessments of risk of bias for each included study.	Page 14; Figure 1; Supplementary Table SROB
**Results of individual studies**	19	For all outcomes, present, for each study: (a) summary statistics for each group (where appropriate) and (b) an effect estimates and its	Table 1; Table 2

		precision (e.g. confidence/credible interval), ideally using structured tables or plots.	
**Results of syntheses**			
**Results of syntheses**	20a	For each synthesis, briefly summarise the characteristics and risk of bias among contributing studies.	Page 15, Lines 229–236; Table 1; Page 14 (risk-of-bias summary)
**Results of syntheses**	20b	Present results of all statistical syntheses conducted. If meta-analysis was done, present for each the summary estimate and its precision (e.g. confidence/credible interval) and measures of statistical heterogeneity. If comparing groups, describe the direction of the effect.	Pages 15–17, Lines 238–322 (qualitative direction-of-effect results; no meta-analysis)
**Results of syntheses**	20c	Present results of all investigations of possible causes of heterogeneity among study results.	Not applicable – no formal heterogeneity analyses were conducted.
**Results of syntheses**	20d	Present results of all sensitivity analyses conducted to assess the robustness of the synthesized results.	Not applicable – no sensitivity analyses were conducted.
**Reporting biases**	21	Present assessments of risk of bias due to missing results (arising from reporting biases) for each synthesis assessed.	Considered within GRADE (publication-bias domain); see Table 4 (Summary of Findings) and Supplementary Table S-GRADE.
**Certainty of evidence**	22	Present assessments of certainty (or confidence) in the body of evidence for each outcome assessed.	Page 17, Lines 333–336; Table 4 (Summary of Findings, GRADE); Supplementary Table S-GRADE.
**DISCUSSION**			

**Discussion**	23a	Provide a general interpretation of the results in the context of other evidence.	Page 18, Lines 247–258
**Discussion**	23b	Discuss any limitations of the evidence included in the review.	Pages 18–19, Lines 422–432
**Discussion**	23c	Discuss any limitations of the review processes used.	Not explicitly discussed.
**Discussion**	23d	Discuss implications of the results for practice, policy, and future research.	Page 19, Lines 404–407
**OTHER INFORMATION**			
**Registration and protocol**	24a	Provide registration information for the review, including register name and registration number, or state that the review was not registered.	Page 3, Lines 105–106 (protocol developed a priori; not registered)
**Registration and protocol**	24b	Indicate where the review protocol can be accessed, or state that a protocol was not prepared.	Page 3, Lines 105–106; Appendix A
**Registration and protocol**	24c	Describe and explain any amendments to information provided at registration or in the protocol.	Not applicable (no registered protocol).
**Support**	25	Describe sources of financial or non-financial support for the review, and the role of the funders or sponsors in the review.	Page 20, Lines 463–464
**Competing interests**	26	Declare any competing interests of review authors.	Page 20, Line 464
**Availability of data, code and other materials**	27	Report which of the following are publicly available and where they can be found: template data collection forms; data extracted from included studies; data used for all analyses; analytic code; any other materials used in the review.	Appendix A and Supplementary materials (search strategy, ROBINS-I tables, GRADE profiles, PRISMA checklist)

From: Page MJ, McKenzie JE, Bossuyt PM, Boutron I, Hoffmann TC, Mulrow CD, et al. The PRISMA 2020 statement: an updated guideline for reporting systematic reviews. BMJ 2021;372:n71. doi: 10.1136/bmj.n71. This work is licensed under CC BY 4.0. To view a copy of this license, visit https://creativecommons.org/licenses/by/4.0/
